# Investigating if changing the wording of a study invitation letter increased recruitment in a project promoting tobacco cessation in a town with high smoking rates

**DOI:** 10.1186/s13011-026-00704-x

**Published:** 2026-01-27

**Authors:** Christina Schell, Alexandra Godinho, John A. Cunningham

**Affiliations:** 1 Institute for Mental Health and Policy Research, Centre for Addiction and Mental Health, Toronto, ON Canada; 2 Humber River Health Research Institute, Humber River Health, Toronto, ON Canada; 3 Department of Psychiatry, University of Toronto, Toronto, ON Canada; 4 Institute of Psychiatry, Psychology and Neuroscience, National Addiction Centre, King’s College London, London, UK

**Keywords:** Reading level, Messaging, Tobacco cessation, Nicotine patches, NRT

## Abstract

**Background:**

Invitation letters can be the first point of contact between research staff and potential participants and a number of modifiable variables have been studied to improve their effectiveness. The aim of this analysis was to determine if lower reading levels and “gain-framed” messages improved recruitment for a RCT that mailed nicotine replacement therapy (NRT) patches for smoking cessation.

**Methods:**

Invitation letters were created which varied in a two-by-two design by reading level and message framing. The letters were randomly mailed to all households in a community selected to receive targeted distribution of NRT patches.

**Results:**

There were no significant differences found in the number of participants enrolled.

**Conclusion:**

Future research investigating factors to improve invitation letters is merited despite current project limitations.

**Trial registration:**

ClinicalTrials.gov NCT04534231.

## Background

High response and enrolment rates increase the likelihood of recruiting representative samples [1], which is important for obtaining valid and reliable data and ensuring the generalizability of results [2]. Investigating methods to improve recruitment is especially valuable for studies involving hard-to-reach groups, such as community samples of people who use substances [2]. One variable that merits attention is written invitation letters. As one of the first points of contact with potential participants, these letters can be instrumental in an individual’s decision to join a study [2]. However, written study material is frequently criticized for its complexity [2] and there is evidence suggesting that participant understanding of study information sheets has decreased over time [3]. 

One factor that contributes to the complexity is language choice. Often documents are written well above the recommended Grade 8 reading level (student age about 13 years) [1]. This impacts the reader’s understanding of important study features, including the purpose, risks, and benefits, which in turn influences attitudes and willingness to participate [2, 4]. Another concern with writing study documents at higher reading levels is that it may create a barrier for recruiting some individuals [4]. According to the Organization for Economic Cooperation and Development (OECD), the literacy skills of nearly half of Canadians falls below what is considered an essential level for an individual to “participate in society, achieve goals, and develop their knowledge and potential” through “accessing, identifying, and processing information from a variety of texts.” [5] To address these concerns, it is generally recommended that documents use simple, plain language; [2] however, while there is evidence that optimally written study documents can aid recruitment [1, 3], other research has reported no effect [1], or effects only in some samples (e.g. depression, cardiovascular disease) [6], but not others (e.g. cancer survivors) [3]. 

Further research is merited by such mixed results; however, readability is not the only aspect of invitation letters that potentially impacts recruitment. Another factor that has been studied but has also produced mixed findings is message framing. In the field of health promotion, the intent of a message can often be stated simply and clearly as a desire to have an individual increase or decrease a behavior. Message framing provides context and tries to increase the likelihood an individual will attempt to change a behavior [7]. Using smoking cessation as an example, the intent of the message is for the individual to quit smoking, but the message may be gain- or loss-framed. Gain-framed cessation messages would include outlining the benefits to health which result from quitting smoking while loss-framed messages would state the harms of continuing to smoke [8]. There is evidence that gain-framed messages are more effective in encouraging prevention behaviors, such as smoking cessation; however, the advantage appears weak [7]. More supporting evidence is needed, as well as, further research investigating possible moderating factors, such as gender, risk perceptions, and/or nicotine dependence [7, 8]. 

To investigate the effect of reading level and message framing on recruitment, four invitation letters were created as part of a randomized controlled trial (RCT). The letters varied in a two-by-two design by reading level (above/below Grade 8) and message framing (gain/loss). The aim was to determine if the content and wording of the letter influenced the number of people that contacted research staff to participate. Based on the literature, we hypothesized that letters written below a Grade 8 reading level and with gain-framed messages would improve recruitment. Furthermore, the letter with both the lower reading level and gain-framed message would have the greatest effect and the letter with both the higher reading level and the lost-framed message would have the least effect.

## Methods

The protocol and outcomes have been published elsewhere [9, 10]. Briefly, municipalities (census sub-divisions) with average smoking rates in the top quartile were identified by reviewing the results of Statistics Canada’s most recent Canadian Community Health Surveys (CCHS) [11]. Based on the participation rate of other projects which distributed NRT patches through postal mail [12] and in order to accommodate the budget of this pilot innovation project, a small, rural community with approximately 7,200 residents was selected from those identified by the CCHS [9]. While this did not result in a randomly selected municipality, it was considered necessary in order to test the effectiveness of a concentrated distribution of NRT patches and ensure that all households in the intervention community could be invited to participate. Addresses in the target community were obtained using an address list available for marketing purposes from Canada Post. The list was licensed for 90-days and over 7,200 letters were sent, one to each household, near the end of October 2020 [9]. 

One resident per household was invited to contact the research team for a free 5-week supply of NRT patches. Individuals were eligible if they were at least 18 years old, smoked 10 or more cigarettes per day for at least three months, and had no health contraindications. The four letter versions were randomly mailed to community addresses. A unique registration code was included to allow staff to identify which letter version was received. Sample text from each version is presented in Table 1 [9]. 

**Table 1:** Sample text from invitation letters combining reading level and message framing

Reading level	Message framing	
	Gain	Loss
Below Grade 8	Adults from [municipality] who smoke are invited to be part of a study. You will get 5-weeks of free nicotine replacement therapy (NRT) patches, if you qualify. If you stop smoking it will benefit your health by preventing problems like lung and other cancers, heart disease and stroke.	Adults from [municipality] who smoke are invited to be part of a study. You will get 5-weeks of free nicotine replacement therapy (NRT) patches, if you qualify. Smoking will harm your health by causing problems like lung and other cancers, heart disease, and stroke.
Above Grade 8	Current adult smokers in [municipality] are invited to participate in a research study and receive a free, 5-week supply of nicotine replacement therapy (NRT) patches. Quitting smoking will benefit your health by preventing problems like lung and other cancers, heart disease and stroke.	Current adult smokers in [municipality] are invited to participate in a research study and receive a free, 5-week supply of nicotine replacement therapy (NRT) patches. Smoking will harm your health by causing problems like lung and other cancers, heart disease, and stroke.

As of July 2023, approximately 450 letters had been returned for various reasons (e.g. empty building, address errors). Due to low enrollment, the study protocol was amended to include other methods of recruitment about two months after mailing the letters. Before this change, 117 participants enrolled with a coupon code indicating they received an invitation letter. This analysis focusses on these participants rather than the total sample (*n* = 123) which was reported elsewhere [10].

Chi-square analyses were conducted to determine if there was any differences in the number of people who requested to participate based on the version of the invitation letter they received. Further chi-square and t-tests were conducted to determine if there were any significant differences in demographic characteristics between the letter groups. All analyses were conducted using SPSS version 27.0 and all the research methods were approved by the ethics review committee at the Centre for Addiction and Mental Health (REB #122/2019).

## Results

Among participants who received letters with less than a Grade 8 reading level, 27 received the gain-framed and 34 received the loss-framed message. Among those who received the letter version with more than a Grade 8 reading level, 27 received the gain-framed and 29 received the loss-framed message. Chi-square analysis revealed no significant differences in the number of participants enrolled by which invitation letter they received (*X*^2^ = 0.183, df = 1, *p* = 0.668). Chi-square and t-tests identified no significant differences by letter condition (Bonferroni adjusted *p* < 0.005) among any demographics (i.e. gender, education, marital status, employment status, household income, age) or clinical characteristics (i.e. number of cigarettes smoked per day, length of time smoking, or the presence of other smokers in the household). Completion of the follow-up interview was also not significantly different between participants in either letter condition.

## Discussion

None of the four invitation letters appears to have made a significant impact on the number of individuals who contacted the research team to enroll, nor do they appear to have had any influence on “who” responded, based on demographic characteristics. Based on the literature, we might have expected more enrollment by participants who received the gained-framed message and/or the letters written at less than Grade 8 reading level; however, the small sample size is a limitation and likely contributes to this null result.

Despite implementing additional recruitment strategies, recruitment primarily relied on postal mail and only a limited number of those invited requested patches. While other methods, such as social media or telephone recruitment, may have resulted in higher response rates, a particular challenge for this project was ensuring participants resided in the intervention community. Another important factor in this low participation rate is possibly the ongoing COVID-19 pandemic. Indeed, the pandemic appears to have had a mixed impact on smoking behavior and cessation attempts in general. Some research has reported increased cessation attempts, while others report increased smoking and still others report no change at all [13, 14]. Our own analysis of Google Trends demonstrated a significant decrease in searches for smoking cessation in Canada during the pandemic compared to the previous two years [15]. If the decreased trend in searches reflects a decreased interest in smoking cessation, it may help explain our challenges recruiting. This possibility is further supported by difficulty experienced during another smoking cessation project undertaken around the same time where the projected recruitment took nearly twice as long as expected [12, 16]. 

Another possible challenge related to the pandemic was the notable increase in scams and fraud. This is common in times of crisis and has been reported by different organizations and countries during the pandemic. For example, 77% of respondents of the Association of Certified Fraud Examiners survey reported observing an overall fraud increase [17]. Likewise, the UK’s Office for National Statistics reported an 85% increase in all cybercrime over the year ending June 2021 compared to the previous year and phishing attacks (i.e. messages delivered by electronic channels to persuade individuals to perform actions for the attacker’s benefit) are estimated to have increased nearly 600% [14]. Indeed, Google alone reported blocking 18 million COVID-19 related phishing attacks in one week in April and 240 million spam messages daily [18]. This uncertainty may have increased suspicions and played a role in some individuals choosing not to participate. Unfortunately, the challenges caused by COVID-19 may have outweighed any improvements gained by a difference in the invitation letters. Another possible limitation of the current project was that there might not have been sufficient perceptual difference between the message framings [1]. Likewise, while a Grade 8 reading level has been recommended in the literature [1], it may not have been appropriate for our target population. Indeed, some researchers have recommended a Grade 5 or 6 reading level [4]. Future projects might consider pilot testing documents with members of the intended population to optimize the different conditions [4] and ensure the overall readability and comprehension of the material.

## Conclusion

While this analysis is somewhat limited, there is still merit in future research investigating the role of reading level and message framing in recruitment and enrollment. More evidence is needed to support existing research that when a behavior has a relatively certain outcome, gain-framed messages tend to be more persuasive [8]. Future work should be directed towards better understanding how moderating variables like gender, risk perception, and/or nicotine dependence interact with framing [8]. This may improve how well a message “fits” a target individual. Making the information feel more relevant may in turn increase the likelihood of the individual attempting to change the desired behavior [8]. 

Future research should also consider other aspects of invitation letters and written material that might play a role in recruitment. For instance, document length has been shown to significantly impact recruitment. Indeed, longer documents are less likely to be read, less likely to be understood, and deter some individuals from participating [3]. Adjusting the length of documents and/or changing the reading level may be challenging however, as some of the information and wording are specified by Research Ethics Boards (REB) for safety and ethical reasons [3, 4]. While the necessity of this information is undeniable, it is also an ethical concern that information be presented in an easily comprehensible manner. Researchers will need to work with REBs to find acceptable compromises where the meaning remains while ensuring comprehension by the target population. Some general recommendations for improving readability for participants is writing at appropriate reading levels for the population of interest, avoiding the use of jargon and technical language, using bullet points, plain language and clear, short sentences [2, 4]. Finally, as online recruitment continues to grow, future work will also be needed to investigate the generalizability of findings between recruitment strategies.

## Data Availability

Data is available from the corresponding author on reasonable request.

## Abbreviations

CND: Canadian Dollar
NRT: Nicotine Replacement Therapy
OECD: Organization for Economic Cooperation and Development
RCT: Randomized Controlled Trial
REB: Research Ethics Board
SD: Standard Deviation
